# Determining attributed factors of hearing handicap in individuals with auditory sensory and neural pathology

**DOI:** 10.1016/j.bjorl.2021.06.003

**Published:** 2021-07-21

**Authors:** Sindhu Parthasarathy, Hemanth Narayan Shetty

**Affiliations:** a All India Institute of Speech and Hearing, Department of Audiology, Mysuru, India; b J.S.S. Institute of Speech and Hearing, Department of Speech and Hearing, Mysuru, Karnataka, India

**Keywords:** Hearing handicap, Auditory neural pathology, Cochlear pathology, Quality of life, Psychoacoustic skills

## Abstract

**Introduction:**

There is a modest relationship between speech perception skills and perceived hearing handicap in individuals with hearing loss. In this study, an attempt is made at linking psychoacoustic results and speech perception skills to understand the subjective handicap and quality of life.

**Objective:**

To investigate how speech perception in noise (signal to noise ratio-50), difference limen frequency, temporal modulation transfer function, hearing handicap inventory for adults, and quality of life in auditory neuropathy spectrum disorder differs from sensorineural hearing loss. Further we attempt to discern attributed factors of hearing handicap in auditory neuropathy spectrum disorder and sensorineural hearing loss.

**Methods:**

A cross-sectional study with comparative and correlational research designs were utilized. Eighty-four participants were grouped into sensorineural hearing loss (n = 49), and auditory neuropathy spectrum disorder (n = 35) was sub-grouped into mild, moderately severe, and severe. We evaluated signal to noise ratio-50, difference limen frequency, and temporal modulation transfer function. In addition, hearing handicap inventory for adults, and quality of life questionnaires were administered.

**Results:**

Mild auditory neuropathy spectrum disorder showed impairment in speech perception and discriminating frequency, which were similar to the severe sensory neural hearing loss. Temporal resolution impairment in auditory neuropathy spectrum disorder mild was significantly higher than in each sub-groups of sensorineural hearing loss. The severity of the hearing handicap in was similar to severe sensorineural hearing loss, quality of life was equally affected in sensorineural hearing loss and auditory neuropathy spectrum disorder. In sensorineural hearing loss, signal to noise ratio-50 was positively related, and quality of life was negatively related to hearing handicap. In auditory neuropathy spectrum disorder, no contributory factors were related to hearing handicap.

**Conclusion:**

Mild auditory neural pathology demonstrates impairment in speech recognition and psychoacoustic skills similar to severe cochlear pathology. In sensorineural hearing loss, hearing handicap is predicted from quality of life and speech perception, but none of the contributory factors predicted hearing handicap in auditory neuropathy spectrum disorder.

## Highlights

• The study investigates the effect of site of lesion on the contributory factors of hearing handicap.
• The study provides insight into how much the mild ANSD subject suffers against their counterparts who have a sensory impairment.
• The finding purports that “mild” ANSD subjects have listening impairment similar to that of “severe” degree of SNHL subjects.

## Introduction

Hearing loss has a significant impact on hearing handicap and eventually affects the quality of life. Iwasaki et al.^1^ reported a mild correlation between hearing handicap and hearing loss in those individuals with sensorineural hearing loss (SNHL) who had a pure tone average of 67 dB. A few studies Alhanbali et al.^2^ and Pedersen and Rosenhall^3^ have reported a mild relationship between speech recognition score and hearing handicap in SNHL. An equivocal result was observed in the relationship between hearing handicap and the degree of hearing loss in SNHL. However, the relationship between hearing handicap and speech understanding in SNHL showed a modest relationship.

Auditory neuropathy spectrum disorder (ANSD) is a type of SNHL. The onset of hearing loss in ANSD at a later part of their life affects speech understanding ability,^4^, ^5^ results in a communication breakdown and, consequently, a hearing handicap. In ANSD, irrespective of the degree of hearing loss and speech perception scores, the hearing handicap was found to be severe.^6^

Psychoacoustics combines the study of acoustics and auditory physiology to determine the relationship between a sound’s characteristics and the auditory sensation that it provokes.^7^ The temporal modulation transfer function is a psychoacoustic task that assesses temporal resolution. A few studies have reported that patients with cochlear hearing loss often showed reduced temporal resolution.^8^, ^9^ Also, participants of ANSD showed less sensitivity to detect modulation amplitude, and the detection threshold worsens as a function of modulation rate.^10^

Difference limen frequency (DLF) is another psychoacoustic test which assesses the ability to detect the smallest change in frequency between the two tones. Goldstein^11^ documented larger DLF in cochlear hearing loss in than normal hearing subjects, suggesting the loss of neural synchrony in the auditory nerve. Even in ANSD cases, abnormal frequency discrimination was observed below 4 kHz, but normal discrimination ability was observed above 4 kHz.^12^ Due to damage in the cochlea or at auditory nerve, the difficulty in understanding speech exacerbated when listening speech in noise contains limited to available amplitude’ or spectral fluctuations where impaired temporal^10^ and spectral^13^ auditory system unable to capture subtle cues for speech perception. Besides, there is a direct relationship between reduced speech perception scores and impaired psychoacoustic skills. In the present study, an attempt is made to link the psychoacoustic measures and speech perception skills to understand subjective hearing handicap. Thus, it is hypothesized that psychoacoustic measures may reliably relate to the hearing handicap in the study participants.

Nordvik et al.,^14^ reported that the majority of studies suggested quality of life (QOL) diminishes as a function of the degree of hearing, and rehabilitation with the hearing aid seems to improve QOL within the first year. However, in individuals with late-onset ANSD, the quality of life is affected due to limited benefit from rehabilitative devices. In addition to the auditory impairment, they also face psycho-social problem.^15^ It is hypothesized that reduced QOL may correlate with the hearing handicap.

The present study is focused on how the perceptual skill (SNR-50), psychoacoustic skills (TMTF and DLF), quality of life, and hearing handicap differed between ANSD and SNHL subjects. If the scores are different among groups, then to what extent auditory neural pathology subjects have impairment against their counterparts who have an auditory sensory pathology in the above-mentioned skills? Further, the relationship between hearing handicap and each of the measurements was ascertained in each group to determine the attributing factors for hearing handicap. It is hypothesized that mild pathology in the inner ear has impairment similar to the severe degree of auditory neural pathology, and each of the measured skills attributes for hearing handicap. The study aimed to investigate the potential attributing factors of hearing handicap in individuals with SNHL and ANSD. The following objectives were formulated: a) To compare each of the measured skills between the SNHL and ANSD group and b) To find the relationship between hearing handicap and measured skills and identify the potential attributing factors for hearing handicap.

## Methods

A cross-sectional study with comparative and correlational research designs was utilized. The study was a part of a research project which was approved by the ethics committee with the reference number SH/CDN/CC/ARF-59/2019-20. A purposive sampling method was used. The procedures involved in the study were explained to the participants, and informed consent was obtained before the data collection. A total of 84 participants were recruited in the study. We grouped into two based on the site of pathology. Group I (n = 49) included participants with confirmed cochlear hearing loss.

Those participants who fulfilled the following conditions were included in the study:

1 Hearing thresholds ranging from 30 dBHL to 90 dBHL in both ears symmetrically.
2 Normal middle ear functioning as indicated by type “A” tympanogram with elevated reflexes (ipsi- and contra-) at 500 Hz to 4 kHz (an octave) or absent reflexes.
3 Absent TEOAEs or DPOAEs based on hearing loss.
4 Tone decay test: patients who heard the tone for one complete minute without a change in loudness.
5 Native speakers of Kannada.

We sub-grouped these participants into three based on degree of hearing loss: mild (n = 10, mean age = 35 years, age range = 19–50 years, mean threshold =34 dB, male = 6 and female = 4), moderately severe (n = 32, mean age = 38 years, age range = 15–62 years, mean threshold = 56 dB, male = 16 and female = 16) and severe (n = 7, mean age = 45 years, age range = 19–60 years, mean threshold = 80 dB, male = 4 and female = 3).

Group II included 35 individuals with confirmed auditory neuropathy spectrum disorder.

All the participants met the following inclusion criteria:

1 They complained of reduced hearing sensitivity and difficulty understanding speech.
2 They met the diagnostic criteria of auditory neuropathy spectrum disorder as per Starr et al.^16^ and Berlin et al.^17^
3 Hearing thresholds ranging from 30 dBHL to 90 dBHL in both ears symmetrically.
4 Normal middle ear status indicated by type “A” tympanogram and robust otoacoustic emission.
5 Impaired neural response reflected in auditory brainstem response and middle ear reflexes were recruited in the study.
6 Native speakers of Kannada.

Further, the ANSD group was sub-grouped into three based on degree of hearing loss mild (n = 19, mean age = 25 years, age range = 49–49 years, mean threshold = 30 dB, male = 7 and female = 12), moderately severe (n = 11, mean age = 24 years, age range = 17–40 years, mean threshold = 60 dB, male = 3 and female = 8) and severe (n = 5, mean age = 33 years, age range = 19–51 years, mean threshold = 76 dB, male = 3 and female = 2).

Exclusion criteria for group I and II: participants having any history of systemic illnesses and complaints of any psychological or neurological problem were not included in the study.

The procedure of data collection included quantitative and qualitative tests. The quantitative tests included behavioral tests and psychoacoustic tests. Behavioral tests comprised of pure tone audiometry, speech perception in noise (SNR-50). Psychoacoustic tests included difference limen frequency (DLF) and temporal modulation transfer function (TMTF). In addition, we assessed hearing handicap inventory and quality of life under the qualitative tests.

1 Quantitative measures:
  A) Behavioral tests:

SNR 50

Stimulus preparation: We prepared speech shaped noise having a spectrum similar to that of a standardized sentence. The procedure given by Shetty and Mendhakar^18^ was adopted to generate speech shaped noise. We have used three lists of standardized Kannada sentences developed by Geetha et al.,^19^ which are phonetically and phonemically balanced. Each sentence in the list is comprised of five target words. We identified the root means square (RMS) for each sentence, and then we added noise at the desired SNR. We mixed the first list of ten sentences with speech shaped noise at different signal to noise ratios ranged from +12 dB to −6 dB SNR in 2 dB step size. The onset of noise was started 500 ms before the beginning of each sentence and continued for 500 ms after the offset of the sentence. A smooth ramp (rise and fall time) was made to the noise using cosine function to avoid unintended effects. We used the following formula to add noise to each sentence. Like the other two lists of sentences, the noise was added at different SNR using a similar procedure as specified earlier. We used the below-mentioned code to generate the desired SNR in Aux Viewer software (version 1).

$$SNR=\text{w}\text{a}\text{v}\text{e}\left(\text{filename}\right)@\;\text{r}\text{m}\text{s}\;>>500\;ramp\;(wave\;("noise")@\;rms,\;20)$$

Ten sentences embedded at different SNRs were randomized. Each sentence was presented at most comfortable level through a loudspeaker at 0° azimuths. We instructed the participants to repeat the sentence heard. We noted the SNR level at which the testing started (L) and the number of correctly recognized target words in each sentence. The total number of target words from all sentences was added (T). We also documented the total number of words per decrement (W) and SNR decrement step size in each sentence (d). The obtained values were substituted to the Spearman–Karber equation, given below, to determine SNR 50%.

50point =L+ (0.5*d) – d (T)/ W

  A) Psychophysical tasks:

We used the Maximum Likelihood Procedure (MLP) to estimate the thresholds for TMTF and DLF. The laptop loaded with MLP was connected to an auxiliary input of audiometer. The output of the audiometer was delivered bilaterally through headphones at the participants’ most comfortable level.

Temporal modulation transfer function: The TMTF stimulus developed by Lorenzi et al.^7^ was adopted. A sinusoidal amplitude modulation applied to the white noise carrier of 1000 ms, as a function of modulation frequency (FM 8 Hz, 64 Hz, and 128 Hz). These frequencies were selected to include both low and high modulation frequencies. We used a three-interval forced-choice method that estimates the modulation depth, m, with the two down one up approach, which accounts for 70.7% correct detection.^20^ Each participant was instructed to identify an interval containing the amplitude-modulation. The modulation depth of each Frequency Modulates (FM) from 0 dB to −30 dB. The step size of m variation was initially 4 dB and was reduced to 2 dB after the first two reversals. The maximum amplitude modulation used was 0 dB (100%), and the minimum was −30 dB (0%).

We obtained the modulation detection threshold (MDT) in dB in terms of the following equation. We utilized the method adopted by Zeng et al.^21^ to calculate peak modulation sensitivity. The peak modulation sensitivity is the lowest modulation depth (in dB) required to identify the modulation from unmodulated white noise. We calculated the peak modulation sensitivity for each frequency modulation. The mean of the last ten reversals in a block of 14 reversals was taken as the threshold of peak modulation sensitivity for that block $MDT=\pi r^{2}20\;\text{l}\text{o}\text{g}\;10\;\text{m}$, where m is the modulation depth in %.

Difference limen frequency: The stimulus developed by Micheyl et al.^22^ was adopted to assess Difference Limen Frequency. We measured DLF for tones of frequencies 500 Hz, 1000 Hz, 2000 Hz, and 4000 Hz (standard frequencies). DLF is defined as the minimum frequency required to differentiate between two tones, which are closely spaced in terms of frequencies. A standard frequency of 500 Hz and complex harmonics tones of 250 ms duration with a cosine ramp of 20 ms was used. The standard frequency tone of 500 or 500 Hz + df Hz was used. Complex tones having the same phase and amplitudes were derived by adding four sinewaves with frequencies corresponding to 2–5 harmonics of a standard frequency (F0) of 500 Hz. Similarly, the procedure was carried out to generate the stimuli of other standard frequencies. For each of the standard frequency, the frequency discrimination threshold was determined based on a 3 AFC procedure with a two down and one up adaptive method. In this method, we presented a set of three stimuli having one standard tone of F0 + df (Hz), and the other two standard pure tones of F0 were presented sequentially. Participants were instructed to identify complex df tone that is different from the other two tones. The target df was reduced by a particular factor after two correct responses and increased by the same factor after one incorrect response. After 12 reversals, the test is stopped. The mean value of df was calculated from the last eight reversal and expressed in Hz.

  A) Qualitative measures

We administered the Kannada version of the Hearing Handicap Inventory for Adults (HHIA)^23^ and the World Health Organization – Quality of Life (WHO-QOL) on each of the participants of the study.

Hearing handicap inventory for adults: The HHIA questionnaire consists of 13 questions in emotional and 12 questions in the social domain, constituting a total of 25 questions. Each question was rated on a three-point rating scale “yes” as 4, “sometimes” as 2, and “no” as 0. The maximum overall score is 100. The maximum number of points for social and emotional subsections is 48 and 52, respectively. A score of 0 implies no handicap, while a score of 100 implies total handicap. A score ranging from 0% to 16% indicates no handicap; a score of 18%–42% indicates mild-moderate handicap. A score of above 44% indicates a significant handicap. We obtained scores for each domain.

World Health Organization — Quality of life: The Kannada version of the World Health Organization – Quality of Life BREF version^24^ was utilized to assess the quality of life. The questionnaire included 26 questions. Each question was rated on a five-point rating scale, and the score ranged from 1 to 5. It has four domains namely physical (maximum score = 35), psychological (maximum score = 30), environmental (maximum score = 40) and social (maximum score = 15) containing 7, 6, 8, 3 questions, respectively. In addition, two questions involve rating the quality of life that reflects the satisfaction of health. In the questionnaire, one of the questions in the social domain did not seem apt to the Indian context. Hence, we replaced the question according to the recommendation by the International Electro-Technical Commission (IEC). We replaced the question about satisfaction about sex life with the relationship with neighbors. We instructed the participants to select an option from 1 to 5 for each of the questions. We calculated the scores of quality of life, health, and total scores for each of the domains.

Experienced audiologists did the evaluation in the department. Later the data for this study was conducted by the research associate in the lab. It took 30 min to perform all the tests on each participant. The basic audiological evaluation and the data collected were on the same day.

## Results

### Comparison of SNR-50 from individuals with SNHL and ANSD

To achieve 50% recognition of speech, the SNR required was less in mild hearing loss (n = 10) than moderately severe (n = 32), followed by a severe degree (n = 7) of SNHL group. The median SNR required to achieve 50% recognition in mild group was −1 dB (SD = 3.86, range = 10), moderately severe was 3 dB (SD = 23.5, range = 23.5), severe group was 4 dB (SD = 14.93, range = 32). A Kruskal Wallis test showed a significant difference between sub-groups for SNR-50 in SNHL (χ^2^(2) = 8.242; *p* = 0.016). Further, we performed a post hoc Mann–Whitney *U* test to check in which sub-groups of SNHL had caused a significant difference in SNR-50. The mild sub-group of SNHL required significantly lesser SNR to achieve SNR-50 than moderately severe (U = 74, Z = −2.546, *p* = 0.011) and severe sub-groups (U = 12, Z = −2.253, *p* = 0.024). Though the SNR required to achieve 50% recognition in noise was less in moderately severe than severe subgroup, it failed to reach a significant difference (U = 83, Z = −1.064, *p* = 0.287). In the case of ANSD, a measurable SNR-50 was obtained from 13/19 participants of the mild sub-group. The median SNR required to achieve 50% recognition in them was 9.5 dB (SD = 10.61, range = 28.90). However, no measurable SNR-50 was obtained from either moderately severe or severe sub-groups of ANSD.

Further, to know the severity of impairment in the SNR-50 between the cochlear and neural site of the lesion, we compared the mild subgroup of ANSD (n = 19) with each of the subgroups of SNHL on SNR-50 using the Mann–Whitney *U* test. It was observed that mild sub-group of ANSD (median = 9.5) required a significantly higher SNR to achieve 50% speech recognition than mild (median = −1; U = 9.50, Z = −3.94, *p* = 0.000) and moderately severe (median = 3; U = 97.50, Z = −4.03, *p* = 0.000) subgroups of ANSD. In addition, though mild sub-group of ANSD (median = 9.5) required higher SNR to achieve 50% recognition than severe (median = 4; U = 54.50, Z = −0.72, *p* = 0.49), it failed to reach significance. It purports that the mild degree of neural pathology in speech recognition has a similar problem with that of the severe degree of cochlear pathology.

### Comparison of difference limen of frequency and modulation detection thresholds in individuals with SNHL and ANSD

The median and SD of DLF at each frequency from sub-groups of SNHL and ANSD are tabulated in Table 1. We carried out a Kruskal Wallis test to check for differences within the sub-groups on frequency resolution in each frequency. It was found that in the SNHL group, there were no differences between the subgroups on DLF at 500 Hz (χ^2^(2) = 2.62, *p* = 0.270), 1000 Hz (χ^2^(2) = 3.141, *p* = 0.208), 2000 Hz (χ^2^(2) = 1.26, *p* = 0.53), 4000 Hz (χ^2^(2) = 2.22, *p* = 0.328). Similarly, for the ANSD group, there were no significant differences between subgroups on DLF at 500 Hz (χ^2^(2) = 5.29, *p* = 0.071), 1000 Hz (χ^2^(2) = 8.022, *p* = 0.051), 2000 Hz (χ^2^(2) = 1.938, *p* = 0.379), 4000 Hz (χ^2^(2) = 2.672, *p* = 0.263).

**Table 1:** Median and standard deviation (SD) of scores of difference limen frequency (DLF) in sensorineural hearing loss and SNHL and auditory neuropathy spectrum disorder (ANSD).

Type	Sub-groups	Domains	500	1000	2000	4000
SNHL	Mild (n = 10)	Median (range)	36.93 (80.04)	57.68 (146.24)	126.01 (283.59)	139.21 (172.10)
		SD	30.28	44.61	91.59	51.21
	Moderately severe (n = 32)	Median (range)	63.86 (135.58)	85.26 (130.99)	156.99 (258.45)	195.83 (150.76)
		SD	134.07	87.22	126.84	202.71
	Severe (n = 7)	Median (range)	45.2 (193.14)	26.97 (182.64)	71.39 (109.8)	215.65 (289.07)
		SD	115.14	322.86	256.15	301.19
ANSD	Mild (n = 19)	Median (range)	79.54 (233.17)	119.4 (471.04)	174.59 (534.79)	202.35 (609.70)
		SD	61.61	106.03	112.36	175.53
	Moderately severe (n = 11)	Median (range)	105.42 (415.38)	254.5 (831.84)	240.24 (555.85)	308.64 (897.66)
		SD	169.17	244.44	158.58	297.09
	Severe (n = 5)	Median (range)	143.91 (498.88)	253.5 (163.52)	179.84 (410.10)	389.55 (248.06)
		SD	197.19	63.77	194.76	104.64

We combined the DLF data at each frequency as there was no significant difference between the sub-groups. This is true in case of SNHL and ANSD. Further a Mann–Whitney *U* test revealed a significantly better frequency resolution in SNHL than ANSD on DLF at 500 Hz (U = 523.5, Z = −3.139, *p* = 0.002), 1000 Hz (U = 448.50, Z = −3.809, *p* = 0.000), 2000 Hz (U = 642.5, Z = −2.076, *p* = 0.038), and 4000 Hz (U = 553.5, Z = −2.872, *p* = 0.004).

**Figure 1:**
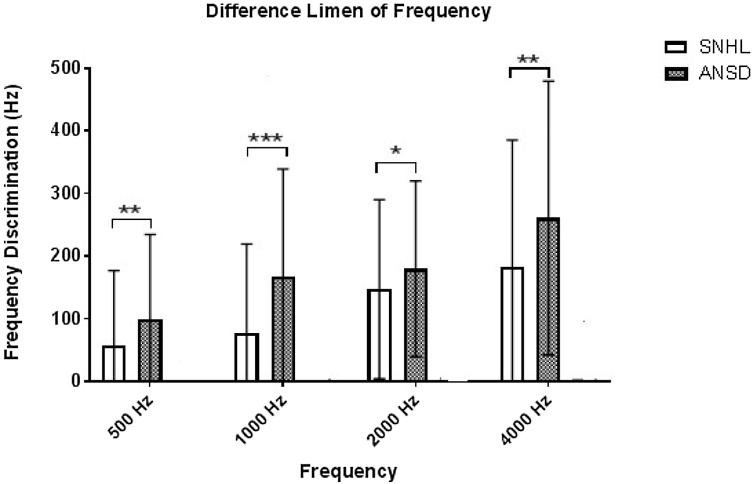
Comparison of difference limen frequency (DLF) in sensorineural hearing loss SNHL and auditory neuropathy spectrum disorder (ANSD).

Further, we compared the mild subgroup of ANSD with each of the sub-groups of SNHL on DLF for each primary tone. A Mann–Whitney *U* test result revealed that the mild subgroup of ANSD required a significantly larger DLF for 500 Hz than each of the mild (U = 42.00, Z = −2.43, *p* = 0.014), moderately severe (U = 14.00, Z = −2.88, *p* = 0.003) and severe (U = 3.00, Z = −2.69, *p* = 0.005) subgroups of SNHL. In addition, a significantly larger DLF was observed in mild ANSD for 1 kHz than mild (U = 32.50, Z = −2.86, *p* = 0.003), moderately severe (U = 7.00, Z = −3.38, *p* = 0.000) and severe (U = 1.000, Z = −2.93, *p* = 0.001) sub-groups of SNHL. Further, a significantly larger DLF was required by the sub-group of ANSD for 2 kHz than mild (U = 69.00, Z = −1.193, *p* = 0.027), moderately severe (U = 26.00, Z = −2.64, *p* = 0.043) and severe (U = 17.00, Z = −0.98, *p* = 0.371) subgroups of SNHL. We observed a similar result for 4 kHz as the mild group of ANSD required larger DLF to discriminate the primary tone from its variable tones than mild (U = 42.00, Z = −2.43, *p =* 0.014), moderately severe (U = 16.00, Z = −0.006, *p* = 0.005) and severe (U = 2.00, Z = −2.81, *p* = 0.003) subgroups of SNHL. It infers that frequency resolution in the mild sub-group of ANSD was affected more than the severe subgroup of SNHL. To sum up, the individuals with ANSD required a significantly higher difference in frequency to differentiate two tones compared to those with SNHL (Fig. 1). Also, frequency resolution in the mild ANSD was affected by more than the severe subgroup of SNHL.

#### Temporal modulation transfer function

The median scores and SD on TMTF in individuals with SNHL and ANSD are tabulated in Table 2. We used the Kruskal Wallis test to check for the difference between sub-groups on scores of TMTF for each frequency. Though the temporal resolution was better in the mild sub-group than moderately severe followed by severe sub-groups of SNHL, it failed to cause significant differences on scores of TMTF at 8 Hz (χ^2^ (2) = 3.529, *p* = 0.129), 64 Hz (χ^2^(2) = 2.81, *p* = 0.245), and 128 Hz (χ^2^(2) = 3.65, *p* = 0.161). Unlike the SNHL group, there was a measurable score on temporal resolution only at 8 Hz and 64 Hz in mild sub-groups of ANSD.

**Table 2:** Median and standard deviation of scores of temporal modulation transfer function (TMTF) in sensorineural hearing loss (SNHL) and auditory neuropathy spectrum disorder (ANSD).

Type	Sub-groups	Domains	8	64	128
SNHL	Mild (n = 10)	Median (range)	−14.7 (10.20)	−8.55 (8.10)	−5.4 (11.40)
		SD	3.73	2.77	3.27
	Moderately severe (n = 32)	Median (range)	−13.35 (17.10)	−8.25 (14.55)	−4.56 (12.15)
		SD	3.91	3.92	3.39
	Severe (n = 7)	Median (range)	−11.85 (15.45)	−5.55 (11.25)	−1.35 (8.40)
		SD	6.52	3.95	3.01
ANSD	Mild (n = 19)	Median (range)	−8.85 (19.65)	−2.25 (15.95)	NA
		SD	5.12	4.62	–
	Moderately severe (n = 11)	Median (range)	NA	NA	NA
		SD	–	–	–
	Severe (n = 5)	Median (range)	NA	NA	NA
		SD	–	–	–

In addition, we compared the mild subgroup of ANSD with each of the sub-groups of SNHL on modulation detection threshold at 8 Hz and 64 Hz. A Mann–Whitney *U* test result revealed that mild subgroup of ANSD required a significantly higher modulation detection threshold for 8 Hz modulation frequency than each of the mild (U = 4.50, Z = −2.27, *p* = 0.021), moderately severe (U = 4, Z = −3.90, *p* = 0.000) and severe (U = 4.00, Z = −2.58, *p* = 0.010) subgroups of SNHL. Similarly, a significant higher modulation detection threshold was observed in mild ANSD for 64 Hz modulation frequency than mild (U = 42.50, Z = −2.41, *p* = 0.014), moderately severe (U = 3.00, Z = −4.18, *p* = 0.000) and severe (U = 3.00, Z = −2.70, *p* = 0.005) sub-groups of SNHL. It infers that the temporal resolution in mild ANSD was affected more than the severe subgroup of SNHL.

### Comparison of hearing handicap inventory in adults with SNHL and ANSD

Table 3 shows that the severity of the hearing handicap was mild to moderate degree in the mild sub-group of SNHL. Whereas hearing handicap was significantly affected in moderately severe and severe sub-groups of SNHL. Also, scores on social and emotional domains were reduced with an increase in the degree of hearing loss. This was true in each domain of hearing handicap inventory. A Kruskal Wallis test was administered to compare the severity of hearing handicap between sub-groups of the SNHL group. The results revealed no differences between the subgroups on the scores of the social domain of HHIA (χ^2^(2) = 3.49, *p* = 0.175), the emotional domain of HHIA (χ^2^(2) = 1.497, *p* = 0.473) and total score of HHIA (χ^2^(2) = 2.757, *p* = 0.252).

**Table 3:** Median and standard deviation (SD) of scores of the hearing handicap inventory for adults (HHIA) in sensorineural hearing loss (SNHL) and auditory neuropathy spectrum disorder (ANSD).

Type	Sub-groups	Domains	Social	Emotional	Total
SNHL	Mild (n = 10)	Median (range)	17 (36)	18 (44)	36 (78)
		SD	13.59	14.83	27.38
	Moderately severe (n = 32)	Median (range)	26 (42)	26 (38)	56 (78)
		SD	12.5	12.07	23.51
	Severe (n = 7)	Median (range)	32 (30)	28 (42)	54 (68)
		SD	12.61	15.99	27.29
ANSD	Mild (n = 19)	Median (range)	34 (44)	34 (38)	68 (78)
		SD	10.39	11.24	20.09
	Moderately severe (n = 11)	Median (range)	32 (26)	34 (42)	60 (68)
		SD	8.54	12.83	20.38
	Severe (n = 5)	Median (range)	40 (14)	34 (24)	72 (30)
		SD	5.76	9.95	11.22

Table 3 shows that hearing handicap is significantly affected irrespective of the degree of hearing loss. Besides, the social domain of HHIA and total score of HHIA was impacted more in the severe subgroup than the mild subgroup, followed by the moderate-severe subgroup of ANSD. In contrast, the emotional domain of HHIA was the same across sub-groups of ANSD. A Kruskal Wallis test revealed no difference between sub-groups on the score of the social domain of HHIA (χ^2^(2) = 2.40, *p* = 0.301) and total score of HHIA (χ^2^(2) = 0.682, *p* = 0.711).

**Figure 2:**
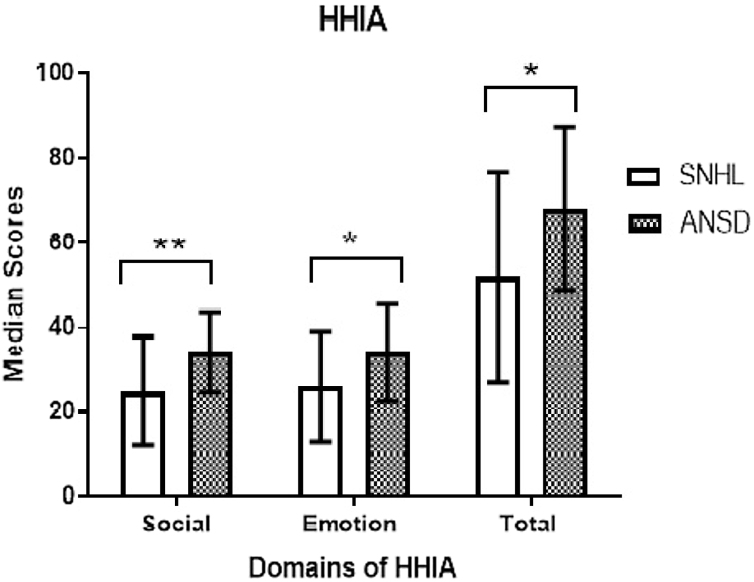
Comparison of scores of each domain of the hearing handicap inventory for adults (HHIA) in sensorineural hearing loss (SNHL) and auditory neuropathy spectrum disorder (ANSD).

The scores in each domain of HHIA obtained between sub-groups were combined in each of the groups as there were no significant differences in them. This was done to compare the scores of each domain of HHIA between SNHL and ANSD. A Mann–Whitney *U* test result revealed a significantly higher severity of hearing handicap in ANSD than SNHL (χ^2^(1) = 6.347, *p* = 0.012) (Fig. 2). This was true in each domain of HHIA, that is, social domain (χ^2^(1) = 7.842, *p* = 0.005) and emotional domain (χ^2^(1) = 4.249, *p* = 0.039).

Further, we compared the mild subgroup of ANSD with each of the subgroups of SNHL on the total hearing handicap index using the Mann–Whitney *U* test. We performed these comparisons to determine the severity of handicap in the mild sub-group of ANSD compared to which subgroups of SNHL. The mild sub-group (median = 68) of ANSD showed a significant higher hearing handicap than mild (median = 36; U = 45.50, Z = −2.27, *p* = 0.021) and moderately severe (median = 56; U = 225.50, Z = −1.67, *p =* 0.049) subgroups of SNHL. Also, though the total hearing handicap in the mild subgroup of ANSD (median = 68) had a higher score on total hearing handicap than the severe sub-group of SNHL (median = 54), it failed to reach significance (U = 55.50, Z = −0.63, *p* = 0.534).

### Comparison of the World Health Organization Quality of Life (WHO–QOL) between individuals with SNHL and ANSD

We performed a Kruskal Wallis test between sub-groups of SNHL on scores of each of the domains on quality of life. The results revealed no significant difference between subgroups of SNHL on quality of life (χ^2^(2) = 0.838, *p* = 0.658), health (χ^2^(2) = 0.014, *p* = 0.993), physical (χ^2^(2) = 0.091, *p =* 0.955), psychological (χ^2^(2) = 0.243, *p* = 0.885), environmental (χ^2^(2) = 1.72, *p* = 0.422), social (χ^2^(2) = 1.623, *p* = 0.444), and total QOL (χ^2^(2) = 0.743, *p* = 0.690) (Table 4). Further, the results of Kruskal Wallis test revealed no significant differences between sub-groups of ANSD on scores of quality of life (χ^2^(2) = 0.468, *p* = 0.792), health (χ^2^(2) = 4.212, *p* = 0.122), physical (χ^2^(2) = 2.16, *p* = 0.340), psychological (χ^2^(2) = 2.383, *p* = 0.304), environmental (χ^2^(2) = 0.082, *p* = 0.960), social (χ^2^(2) = 0.006, *p* = 0.997), total QOL (χ^2^(2) = 0.130, *p* = 0.937) (Table 4).

**Table 4:** Median and standard deviation (SD) of scores of quality of life (QOL) in sensorineural hearing loss (SNHL) and auditory neuropathy spectrum disorder (ANSD).

Type	Sub-groups	Domains	Social	Emotional	Total
SNHL	Mild (n = 10)	Median (range)	17 (36)	18 (44)	36 (78)
		SD	13.59	14.83	27.38
	Moderately severe (n = 32)	Median (range)	26 (42)	26 (38)	56 (78)
		SD	12.5	12.07	23.51
	Severe (n = 7)	Median (range)	32 (30)	28 (42)	54 (68)
		SD	12.61	15.99	27.29
ANSD	Mild (n = 19)	Median (range)	34 (44)	34 (38)	68 (78)
		SD	10.39	11.24	20.09
	Moderately severe (n = 11)	Median (range)	32 (26)	34 (42)	60 (68)
		SD	8.54	12.83	20.38
	Severe (n = 5)	Median (range)	40 (14)	34 (24)	72 (30)
		SD	5.76	9.95	11.22

**Figure 3:**
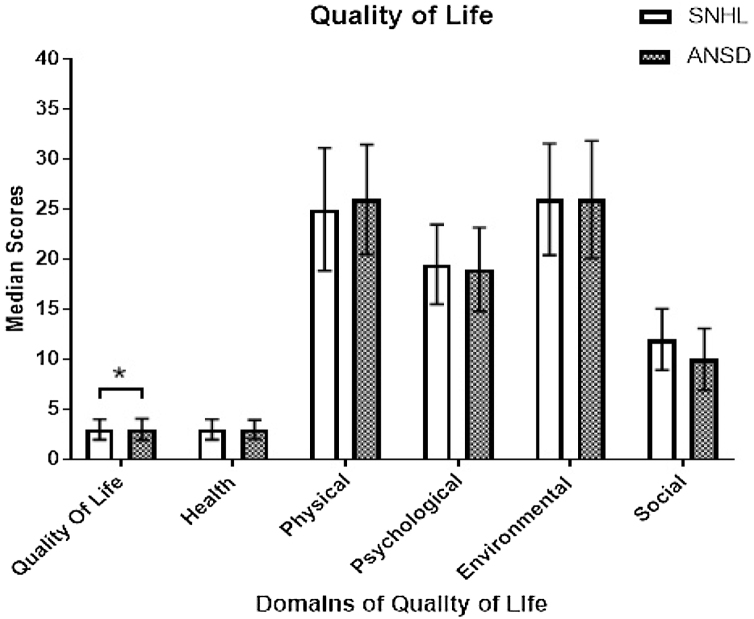
Comparison of scores of domains of quality of life in sensorineural hearing loss (SNHL) and auditory neuropathy spectrum disorder (ANSD).

We combined the scores in each domain of quality of life obtained between sub-groups of SNHL, as there were no significant differences in them. Similarly, it was done for ANSD subgroups. Further, we compared the quality of life between SNHL and ANSD groups. The total score of quality of life was affected more in ANSD than SNHL (Fig. 3), which was found significant (χ^2^(1) = 4.454, *p* = 0.035). In addition, though each domain of quality of life was affected more in ANSD than SNHL, it failed to reach significance in health (χ^2^(1) = 0.075, *p* = 0.785), physical (χ^2^(1) = .238, *p* = 0.626), psychological (χ^2^(1) = 0.238, *p* = 0.626), environmental (χ^2^(1) = 0.644, *p =* 0.422), social (χ^2^(1) = 2.899, *p* = 0.089) and total score of quality of life (χ^2^(1) = 0.486, *p* = 0.486).

We compared the mild subgroup of ANSD with each subgroup of SNHL on the total score of quality of life using the Mann–Whitney *U* test. We performed these comparisons to know the severity of quality of life in the mild sub-group of ANSD compared to which subgroups of SNHL. Though the quality of life was worse in the mild sub-group of ANSD than ieach of the sub-groups of SNHL, it failed to reach significance.

### Relationship between hearing handicap and attributing factors

The attributing factors such as hearing thresholds, speech perception in quiet, SNR-50, modulation detection thresholds, difference limen of frequency, and quality of life in individuals with SNHL and ANSD were utilized to correlate with hearing handicap. This relationship was performed separately for SNHL and ANSD. We performed a Spearman's sign rank correlation coefficient between hearing handicap scores and attributing factors. In individuals with SNHL, we found a significant mild degree of the positive correlation between the total hearing handicap scores and SNR 50 (r = 0.327, *p* = 0.022). In addition, we found a significant mild degree of negative correlation between total hearing handicap and a total score of quality of life (r = −0.382, *p* = 0.006). However, no correlation was found between total hearing handicap scores and an average of pure tone averages (r = 0.259, *p* = 0.069)., DLF at 500 Hz (r = 0.102, *p* = 0.480), 1000 Hz (r = −0.002, *p =* 0.989), 2000 Hz (r = −0.052, *p* = 0.722), 4000 Hz (r = 0.062, *p* = 0.670); and TMTF scores at 8 Hz (r = 0.084, *p* = 0.562), 64 Hz (r = 0.008, *p* = 0.957) and 128 Hz (r = −0.079, *p* = 0.585).

In individuals with ANSD, no correlation was found between hearing handicap and any of the attributing factors: average of pure tone averages (r = −0.024, *p* = 0.892), SNR 50 (r = −0.073, *p =* 0.676), DLF at 500 Hz (r = 0.243, *p =* 0.160), 1000 Hz (r = 0.171, *p* = 0.325), 2000 Hz (r = −0.104, *p =* 0.551), 4000 Hz (r = 0.010, *p* = 0.957) and TMTF scores at 8 Hz (r = 0.157, *p* = 0.368), 64 Hz (r = 0.148, *p* = 0.397), and 128 Hz (r = −0.071, *p =* 0.921). However, there was a significant mild degree of negative correlation between total handicap scores and total quality of life (r = −0.365, *p* = 0.31).

## Discussion

### Speech perception in noise

Individuals with sensorineural hearing loss required higher SNR as a function of the degree of hearing loss to obtain 50% speech recognition in noise. It indicates that SNHL hearing-impaired individuals exhibit greater susceptibility to noise with an increase in the degree of hearing loss^25^ and thus require higher SNRs to understand speech, suggesting secondary distortion due to impaired temporal and spectral resolution.^26^ Thus, it is reasonable to assume that the increase in the degree of hearing loss requires high SNRs to get the contextual cues in the sentences.

The combination of temporal impairment as a function of the severity of hearing loss^27^ and alteration of temporal characteristics of sentences by speech-shaped noise degraded the recognition^28^ even with an increase in SNRs. The result of the study is in consonance with the research study of Stach,^26^ who reported that speech perception abilities are in proportion to their pure-tone hearing loss. This is not true in the case of ANSD participants. The SNR-50 was achieved at higher SNR in the mild-subgroup of ANSD than SNHL. There was no measurable SNR-50 in moderately severe and severe sub-groups of ANSD when SNR was set at 30 dB SNR in the speech recognition test. The results of the present study are in consonance with the previous studies Starr et al.,^29^ who reported a bare minimum score or no measurable speech perception despite ample audibility of sound.

### Temporal modulation transfer function

The temporal processing in ANSD reflected in TMTF is impaired irrespective of the degree of hearing loss for low modulation frequency. These results are in consonance with the results obtained by Narne.^30^ The main characteristic of auditory neuropathy is a significant temporal impairment, who are unable to capture the modulation depth of the envelope in sentences and introduces spurious modulations, which obscures the relevant speech modulations.^30^

Furthermore, ANSD subjects are unable to detect the spatial changes in the excitation pattern along the basilar membrane^31^ due to leakage of signal to neighboring fibers. Complete or partial loss of myelin in the auditory nerve has a significant effect on the generation and propagation of action potentials, as there is an increase in membrane capacitance and a decrease in membrane resistance.^32^

In SNHL it was observed that with an increase in the severity of hearing loss, the TMTF are affected more in a higher degree of hearing loss than their counterpart. Thus, even with an increase in SNRs, the sampled populations of moderately severe and severe subjects of SNHL partly capture the cues for speech perception in noise.

### Frequency discrimination task

Frequency discrimination cue is the basis for speech understanding in noise. In both SNHL and ANSD participants required a larger DLF as a function of increased primary frequency tones and degree of hearing loss. The ANSD participants required a larger DLF than SNHL due to the impaired in the phase-locking ability for discriminating low-frequency primary tones^33^ and altered spatial changes in the excitation pattern along the basilar membrane for discriminating high-frequency primary tone.^31^ Thus it was observed that the severity of impairment in discriminating frequency for the mild subgroup of ANSD was almost similar to that of the “severe” sub-group of SNHL.

### Quality of life

Social isolation, the decline in social activities, and emotional distress make them rate “Not at all” and “somewhat” in each subscale of QOL in both ANSD and SNHL participants. Thus, irrespective of the degree of hearing loss and site of pathology, the QOL is equally affected. This is because the study participants might have reduced self-esteem in social skills. Besides, hearing loss and low coping strategies in them while communication contributes to having an impaired quality of life.

### Hearing handicap

The emotional and social skills of HHIA in ANSD were significantly affected than SNHL. This could be due to a sudden onset of hearing loss in ANSD made them undergo stress which renders them unable to communicate as well as previously. In ANSD, functionality is reduced due to a lack of rehabilitation management strategy, which impacted everyday life, causing loneliness, social isolation, dependence, and frustration, and communication impairment. Thus, the severity of the hearing handicap in the mild sub-group of ANSD was similar to the “severe” sub-group of SNHL.

The participants of SNHL showed a significant increase in hearing handicap in those subjects who took higher SNR in dB required for 50% speech recognition and rated low in quality of life. Individuals with SNHL decipher the meaning of a sentence in higher signal to noise ratio because of the audibility of speech over the noise. Unfortunately, in a daily listening scenario, they may not have experience always an optimum signal to noise ratio to lessen their hearing handicap and quality of life. Thus, the sampled population of SNHL subjects rated increased hearing handicap and reduced QOL in those individuals who wanted higher SNR to recognize 50% speech.

Furthermore, in ANSD, the hearing handicap was not related to the test measures taken up in the present study (TMTF, DLF, SNR-50, and QOL). It purports that the hearing handicap is equally affected irrespective of scores in the measured test from the samples of ANSD participants. This is because they tend to compare the present listening problem with their listening abilities prior to the hearing loss.

### Effect of aging

Deterioration of the temporal processing begins by forth decade of life in individuals with normal hearing.^34^ With respect to the frequency discrimination too, the age-related changes for difference limen start after 40 years of age.^35^ Though the literature purports that the temporal resolution deteriorates with hearing threshold levels and not with age,^36^ the effect of aging could be seen in individuals with hearing loss too. However, in the present study the mean age of all the subgroups was less than 40 only. The mean age for the SNHL group is 35.44 years and SD is 12.57. Similarly, the mean age for the ANSD group is 26.4 years and SD is 9.41 years. But the effect of aging could be more in the SNHL group as there were 16 participants above the age of 40 years, while there were just 2 participants in the ANSD group of age above 40 years. But irrespective of the age component, the issues in processing can be attributed to the neural pathology, as the deficit which the individuals with ANSD experience is much more severe than individuals with SNHL.

### Overall discussion

The mild subgroup of ANSD participants required significantly higher SNR to achieve 50% recognition of speech than the mild subgroup of SNHL. However, there was no measurable SNR for 50% recognition in moderately severe and severe sub-groups of ANSD. In addition, the mild ANSD sub-group has a speech perception impairment similar to that of the severe sub-group of SNHL. Larger DLF was required as a function of frequency. This was true for each of the sub-groups of SNHL and ANSD. A significantly larger DLF was required in ANSD than SNHL for each frequency. Also, the severity of impairment in discriminating frequency in the mild subgroup of ANSD was similar to the severe sub-group of SNHL. In TMTF, there was no measurable modulation detection threshold for moderately severe and severe groups of ANSD in each of the modulation rates. Whereas mild sub-group of ANSD required a higher modulation detection threshold than the mild sub-group of SNHL at 8 Hz and 64 Hz, respectively. Besides, temporal resolution impairment in the mild sub-group of ANSD was significantly higher than in each of the sub-groups of SNHL. The hearing handicap was more significantly affected in ANSD than SNHL. The severity of the hearing handicap in the mild sub-group of ANSD was similar to the severe sub-group of SNHL. Quality of life was equally affected in both SNHL and ANSD groups. In the SNHL group, the SNR-50 was positively related, and QOL was negatively related to hearing handicap. Speech perception and quality of life were the contributory factors for hearing handicap in the SNHL group. Whereas, in ANSD, none of the factors are related to hearing handicap.

## Conclusion

Speech perception in noise, psychoacoustic skills, hearing handicap, and QOL was significantly affected more in ANSD than SNHL groups. Also, the severity of the mild sub-group of ANSD was similar to those participants of the severe sub-group of SNHL in each of the behavioral skills, psychoacoustic skills, hearing handicap, and QOL assessed. Further, the hearing handicap in SNHL was attributed to SNR-50 and quality of life. In ANSD, the hearing handicap is not related to any of the attributor factors undertaken in the study. The study provides insight into how much the mild ANSD subject suffer against their counterparts who have a sensory impairment. The finding purports that “mild” ANSD subjects have listening impairment similar to that of “severe” degree of SNHL subjects. The limitation of the study is the fact that in the sample of each group, we were unable to categorize their hearing handicap or QOL based on the scores of tests measured in the present study. This is because the sampled population rated the same degree of hearing handicap or QOL irrespective of the degree of hearing loss.

## The implication of the study

This study provides insight into how much the mild ANSD subject suffers against their counterpart who has a sensory impairment. The finding purports that “mild” ANSD subjects have listening impairment similar to that of “severe” degree of SNHL subjects.

## Funding

This study was funded by the All India Institute of Speech and Hearing, Mysuru.

## Conflicts of interest

The authors declare no conflicts of interest.
